# Impact of cervical screening on cervical cancer mortality: estimation using stage-specific results from a nested case–control study

**DOI:** 10.1038/bjc.2016.290

**Published:** 2016-09-15

**Authors:** Rebecca Landy, Francesca Pesola, Alejandra Castañón, Peter Sasieni

**Affiliations:** 1 Centre for Cancer Prevention, Wolfson Institute of Preventive Medicine, Bart's & The London School of Medicine, Queen Mary University of London, Charterhouse Square, London EC1M 6BQ, UK

**Keywords:** cervical cancer, screening, mortality, impact of screening on mortality, estimating impact of screening

## Abstract

**Background:**

It is well established that screening can prevent cervical cancer, but the magnitude of the impact of regular screening on cervical cancer mortality is unknown.

**Methods:**

Population-based case–control study using prospectively recorded cervical screening data, England 1988–2013. Case women had cervical cancer diagnosed during April 2007–March 2013 aged 25–79 years (*N*=11 619). Two cancer-free controls were individually age matched to each case. We used conditional logistic regression to estimate the odds ratio (OR) of developing stage-specific cancer for women regularly screened or irregularly screened compared with women not screened in the preceding 15 years. Mortality was estimated from excess deaths within 5 years of diagnosis using stage-specific 5-year relative survival from England with adjustment for age within stage based on SEER (Surveillance, Epidemiology and End Results, USA) data.

**Results:**

In women aged 35–64 years, regular screening is associated with a 67% (95% confidence interval (CI): 62–73%) reduction in stage 1A cancer and a 95% (95% CI: 94–97%) reduction in stage 3 or worse cervical cancer: the estimated OR comparing regular (⩽5.5yearly) screening to no (or minimal) screening are 0.18 (95% CI: 0.16–0.19) for cancer incidence and 0.08 (95% CI: 0.07–0.09) for mortality. It is estimated that in England screening currently prevents 70% (95% CI: 66–73%) of cervical cancer deaths (all ages); however, if everyone attended screening regularly, 83% (95% CI: 82–84%) could be prevented.

**Conclusions:**

The association between cervical cancer screening and incidence is stronger in more advanced stage cancers, and screening is more effective at preventing death from cancer than preventing cancer itself.

Service evaluation of cancer screening is essential to monitor effectiveness, to identify areas of good practice and areas where improvements can be made (Sasieni and Cuzick, 2001). The evaluation of programme effectiveness is usually undertaken by linking data on incidence and mortality from cancer registries to individual-level information regarding screening uptake and results (NHS Cervical Screening Programme, 2006).

The National Health Service Cervical Screening Programme in England was established in 1988. In the early 1990s, a cervical cancer audit was initiated to evaluate the effectiveness of this programme in preventing cervical cancer. Whereas much has been published on the impact of the programme on the incidence of cervical cancer (Sasieni *et al*, 2003; Sasieni *et al*, 2009), the effect of screening on cervical cancer mortality is not known; as mortality is the most demanding end point, it requires large numbers of patients with long follow-up to ensure adequate power (Cuzick *et al*, 2007). For cancers with moderate or good survival, it is highly desirable to have surrogate end points that can reliably predict mortality reductions several years earlier.

Several authors have estimated the effect of cervical screening on cervical cancer mortality using mortality trends (Dickinson *et al*, 1972; Laara *et al*, 1987; Sasieni and Adams, 1999). However, trends are prone to bias and can only reflect population-level mortality. Further, they do not directly estimate the efficacy in screened women. In this paper, we use the data from a case–control study of cervical cancer incidence, containing individual-level screening histories, to estimate the preventive effect of regular screening on cervical cancer of each FIGO (Fédération Internationale de Gynécologie et d'Obstétrique) stage.

## Materials and methods

### Study population

We use data from the Audit of Invasive Cervical Cancers (NHS Cervical Screening Programme, 2006), a population-based case–control study in England that has been estimated to include ∼90% of all cervical cancers with better completeness under age 65 years. Cases were all women who had cervical cancer (ICD-10 C53) diagnosed in England between April 2007 and March 2013 at age 25–79 years, and were registered with an NHS general practitioner (GP). All women, except for those with a prior hysterectomy, registered with an NHS GP who did not have cervical cancer at the time of the case's diagnosis were eligible as a control. Using a computer programme, two controls were individually matched on age and area of residence to each case. Very occasionally, only one control could be found. Data were collected on all selected controls, removing the possibility of participation bias.

Prospectively recorded cervical screening data were abstracted from routinely recorded cervical cytology records held on the Cervical Screening Call/Recall System, and were therefore not subject to recall bias. These records include all NHS (and many private provider) smears taken in the United Kingdom since 1988. Local NHS staff linked the screening data to the cases and controls, and the data were pseudonymised locally before being transferred for cleaning and analysis. Guidelines on the collection of data for this audit and details of the design have been published previously (Sasieni *et al*, 2003; NHS Cervical Screening Programme, 2006; Sasieni *et al*, 2009). Routine screening in England is offered 3 yearly to women aged 25–49 years and 5 yearly at ages 50–64 years.

We excluded women diagnosed under age 25.5 years for most analyses because there is a peak in prevalent cancer diagnosis at age 25 (24.75–25.5) years when women are first screened (Castanon *et al*, 2013). These prevalent cancers are primarily early stage (76% stage 1A) and very few are fatal. Inclusion of these cancers skews estimation of the effect of screening on cancer incidence.

### Statistical analyses

We assumed that all stage 1A cancers in the audit data set would have the stage recorded. Therefore, cases with unknown stage were assumed not to be stage 1A, but otherwise to be missing at random (conditional on age). Cases with missing stage were therefore proportionally allocated to stages 1B, 2, or 3+, with the proportion in each of these stages determined by the prevalence of the stage in each age group.

As age- and stage-specific mortality data are not available for England, we used stage-specific 5-year relative survival from the former Anglia Cancer Network, 2002–2006 (Cancer Research UK, 2014), and modified it using stage- and age-specific statistics from SEER (Kosary, 2007) as follows: hazard ratios for different age groups conditional on stage were calculated from SEER. These were then applied to the Anglia survival and normalised (with a stage-specific hazard ratio) so that the marginal stage-specific survival matched that reported by Anglia. The age- and stage-specific survival was then renormalised (with an age group-specific hazard ratio) to ensure that the marginal 5-year relative survival in each age group matched those reported for England as a whole (Office for National Statistics, 2013). The overall 5-year relative survival for Anglia between 2006 and 2010 (across all stages) was 69.6% among women aged 15–99 years, compared with 67.4% for England and Wales between 2010 and 2011, and 71.5% in SEER between 1988 and 2001 (aged 20+ years). To estimate the impact of screening on the risk of cancer diagnosis at each stage, each woman's maximum screening interval was calculated. This was defined as the longest period during the screening window (see below) in which there were no adequate smears (i.e., test result not reported as ‘inadequate' that equates to ‘unsatisfactory' in other cytology systems). These were then used to classify women as: regularly screened, irregularly screened, very irregularly screened, or not (or poorly) screened (Table 1). For women aged ⩾65 years, the screening window was ages 50–64 years. For women aged 35–64 years, the window was 15 years before case diagnosis. For women aged 25–34 years, a slightly different definition of screening regularity was used (Table 1). For controls, the date of diagnosis was taken to be their matched case's date of diagnosis. Conditional logistic regression models were used to estimate the odds ratios (OR) for developing each stage of cancer by maximum screening interval.

The numbers of cancers and excess deaths within 5 years of diagnosis were estimated: (i) under current screening; (ii) in the absence of screening; and (iii) assuming everyone was regularly screened. For each FIGO stage, the numbers of cancers that would occur in the absence of screening were estimated using equation (1).


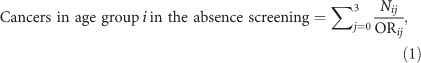


where *N*_*ij*_ is the number of (stage-specific) cases (in the audit, adjusted for those with unknown stage and under-reporting compared to ONS cancer registrations) in age group *i* with screening category *j* (see Table 1 for screening category definitions, with 0 corresponding to the ‘not screened' category) and OR_*ij*_ is the OR of developing (stage-specific) cancer for women in age group *i* in screening category *j*, compared with a baseline category of ‘never screened'. There are three screening categories in addition to the baseline category (i.e., OR_*i0*_); by definition OR_*i0*_=1.

The excess number of deaths within 5 years of diagnosis of a specific stage of cancer in the absence of screening was estimated using equation (2).


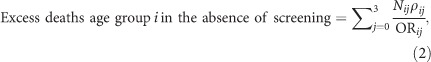


where *ρ*_*ij*_ is one minus the 5-year relative survival in age group *i* with screening category *j* (for cancers of the particular stage). As stage 1A cancers are unlikely to be diagnosed in the absence of screening, all stage 1A cancers in never-screened women are assumed in the main analysis to have stage 1B survival. This assumption is relaxed in a sensitivity analysis.

We converted the effect of screening at different ages into absolute numbers of cervical cancer deaths. We assume that the age intervals of death are shifted by 5 years, for example, 25–34 years at diagnosis corresponds to 25–39 years at death, and 35–49 years corresponds to 40–54 years.

For the estimated deaths in the absence of/regular screening within age groups, the relative risks (RRs) were multiplied by the observed deaths. The RRs were estimated from the sum of the estimated deaths in each age group when looking at all age groups combined. The confidence intervals (CIs) for these RR were obtained using the appropriately weighted sum of the variance of the individual age groups. Deaths from cervical cancer at all ages were included in this analysis to explore the effect of the screening programme in the population as a whole rather than the effect of screening in the target age group; however, we assume that screening from age 25 to 64 years will not prevent deaths from cancers diagnosed at age under 25 years or age over 79 years.

For each age group and a particular level of screening, the RR for incidence (mortality) was indirectly calculated by summing the estimated number of cancers (excess deaths) diagnosed across all stages, and dividing by the estimated number of cancers (deaths) in the absence of screening. For comparison, the directly calculated OR for incidence was calculated using a conditional logistic regression model, including all cancers diagnosed in the relevant age group, regardless of stage. As the indirect estimates are weighted sums of the (age and) stage-specific ORs, CIs were estimated using the delta method (for the variance of the log RR), with an (appropriately) weighted sum of the estimated (age and) stage-specific variances (Armitage *et al*, 2002).

We carried out a number of sensitivity analyses to test the effect of (i) changing the screening window; (ii) assigning cancers with missing stage using a number of criteria; (iii) applying stage 1A mortality to the 1A cancers in the absence of screening scenario; (iv) including women aged 24.5–25.5 years in the analysis. For the sensitivity analyses, we present the OR comparing ‘never'-screened women to the current level of screening in the population (i.e., control women) for comparison with Table 4.

Analyses were carried out using Stata 13 (StataCorp, 2013).

## Results

**Figure 1:**
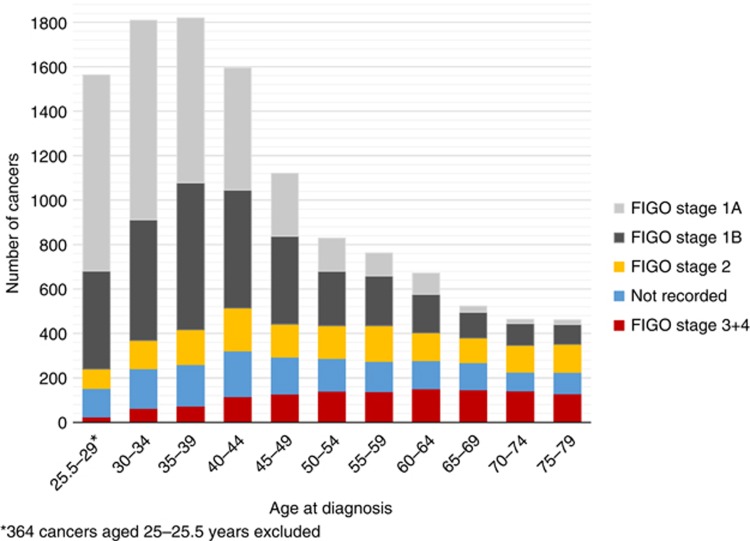
**Number of cervical cancers by age group and FIGO stage.**

A total of 11 619 cases of cervical cancer diagnosed aged 25.5–79 years were included in this study. For 2008–2012, the number of cancers in women aged 25–79 years in the audit was 89.2% of those reported in the Cancer Registry (Office for National Statistics, 2007–2012). FIGO stage was recorded for 10 040 cancers (86.4%). Of those with a known stage, over a third were stage 1A (37.5%), with a further 35.1% diagnosed at stage 1B. Overall, 39.0% of the cancers were diagnosed at age 35–49 years, with 29.0% diagnosed aged 25.5–34 years and 12.5% aged 65–79 years. The stage distribution varied by age (Figure 1); 58.1% of women diagnosed aged 25.5–34 years (with stage recorded) were diagnosed at FIGO stage 1A and only 9.8% were diagnosed at stage 2+ whereas two-thirds (67.5%) of women diagnosed aged 65–79 years were diagnosed at stage 2+.

Table 2 shows the ORs of a cervical cancer diagnosis by age, stage, and maximum screening interval. At all ages, screening is associated with a reduction in cancer diagnoses. Screening at age 50–64 years is associated with a reduced cervical cancer incidence at ages 65–79 years. The negative association between screening and cervical cancer is stronger for more advanced stages of cancer. Although stage 1A cancer is generally screen detected (while still asymptomatic), screening at all ages is associated with a lower odds of being diagnosed (after age 25.5) with stage 1A cancer.

The estimated 5-year case fatalities (i.e., one minus 5-year relative survival) in England by age and stage are shown in Table 3. They depend heavily on stage at diagnosis and to a lesser extent on age (for a given stage). Five-year case-fatality rates increase with age within each stage, and are highest for women diagnosed aged 70–79 years.

We estimate that there would be 2.53 (95% CI: 2.39–2.68) times as many cancers diagnosed aged 25–79 in the absence of screening, and a third less (RR=0.66, 95% CI: 0.64–0.67) if everyone was regularly screened. The largest impact of changing screening practices on cancer incidence rates is for women aged 50–64 years, where the incidence rate would be over four times higher with no screening (RR=4.15, 95% CI: 3.63–4.74), and less than half (RR=0.48, 95% CI: 0.46–0.51) if everyone was regularly screened. Table 4 shows the mortality rates (incidence multiplied by one minus the 5-year relative survival) in the absence of screening and under regular screening compared with current screening (assuming the observed associations are causal). In the absence of screening, mortality would be four times higher (RR=4.13, 95% CI: 3.59–4.75) for women aged 35–49 years and over five times higher (RR=5.30, 95% CI: 4.36–6.44) for women aged 50–64 years (Table 4). Conversely, if everyone was regularly screened, mortality would be less than half what it currently is (RR=0.42, 95% CI: 0.38–0.47) for women aged 35–49 years, and be reduced by two-third (RR=0.35, 95% CI: 0.33–0.37) for women aged 50–64 years at diagnosis.

Results from the sensitivity analysis (Supplementary Table) suggest that including cancers in women aged 24.5–25.5 years reduced the impact of screening in women under age 35 years by 21% for incidence (from 1.29 to 1.02) and by 16% for mortality (from 2.20 to 1.84). The impacts of other changes were much less. When we assume that stage 1A cancers could be ‘opportunistic' findings and would have the same fatality rates whether screen detected or opportunistic, the mortality ORs were reduced by between 0.8 and 18.2% depending on age, with the greatest effect seen in young women where stage 1A cancer is most common. Similar results were observed for the sensitivity analyses varying the screening window (i.e., 15-, 12-, or 8-year windows and looking only at the two preceding screening intervals) and the missing stage allocation (no reallocation, all stage 2, all stage 3+, and missing at random from stages 1A, 1B, 2, and 3+). In all analyses, the ratio of the mortality OR to the ones reported in Table 4 is between 0.66 and 1.08. The biggest difference (OR reduced from 5.3 to 3.5) was in women aged 50–64 years when considering an 8-year screening window. This suggests that screening continues to have some impact on cervical cancer mortality at ages 50–64 years for >8 years.

The methodology used to estimate the association between screening and mortality relies on the ability to estimate the stage-specific associations and to combine these to obtain an overall association. We test the robustness of this approach by comparing the OR for cervical cancer (incidence) obtained directly without reference to stage and the RRs obtained by combining stage-specific estimates. The two methods produced very similar results for both point estimates and CIs (Table 5). The association between screening and cancer was similar for women aged 35–64 years and 65–79 years, even though the screening interval considered for women diagnosed aged 65–79 years was based on their screening when aged 50–64 years. The association between screening and 5-year cervical cancer mortality is stronger than with cancer incidence for women aged 25.5–64 years. Among women aged 35–64 years, the estimated ORs comparing regular (⩽5.5 yearly) screening to no (or minimal) screening is 0.18, 95% CI: 0.16–0.19 using our approach (identical to 0.18, 95% CI: 0.16–0.20 using the direct method) for cancer incidence and 0.08 (95% CI: 0.07–0.09) for mortality.

In England, there are an average of 796 deaths a year (2011–2014 average) from cervical cancer in women of all ages (Office for National Statistics, 2015). It is estimated that screening currently prevents 69.7% (95% CI: 66–73%) of cervical cancer deaths. However, if everyone attended screening regularly 82.9% (95% CI: 82–84%) of deaths could be prevented (i.e., half of deaths currently occurring could be prevented). Applying the RRs in Table 4 to the observed number of deaths in each age group, we estimate that there would be an additional 1827 deaths per year from cervical cancer in the absence of screening, and a further 347 deaths per year could be prevented if everyone attended screening regularly between ages 25 and 64 years.

## Discussion

It is generally accepted that quality-assured cervical screening reduces cervical cancer incidence. We have shown that regular cervical screening in England is associated with lower cervical cancer incidence, and that the strength of association increases with age and advancing FIGO stage. Assuming associations are causal, the mortality rate would be 5.3 times higher (95% CI: 4.4–6.4) in the absence of screening or 65% lower (95% CI: 63–67%) if everyone was regularly screened at ages 50–64 years. In summary, if all women attended screening regularly, we estimate that the crude mortality for women aged 25.5–79 years would be half the current (95% CI: 0.48–0.52), whereas it would be 3.6 (95% CI: 3.3–4.0) times higher in the absence of cervical screening. The effect on mortality is greater than the effect on cancer incidence, as cervical screening downstages cancers (to stages with improved survival) as well as preventing them.

As far as we are aware, this is the first study to estimate the impact of cervical cancer screening on mortality using an incidence-based case–control study and stage-specific survival. Combining stage-specific incidence and survival to estimate mortality is not new; it was proposed by Cuzick *et al* (Cuzick *et al*, 2007) as an appropriate way in which to predict mortality in cancer screening trials. The combination of stage-specific results to obtain marginal ORs is novel, but we have shown that it works well in these data (Table 5). We have also shown that RRs calculated from a case–control study of incident cancer (with stage information recorded) can be combined with external survival data to estimate the association between a risk factor and cancer mortality.

As we did not have age- and stage-specific survival for England, we have used SEER data for the joint dependence on stage and age, adjusted so the marginals for age-specific survival matched English estimates. We naively equate one minus the 5-year relative survival with the probability of dying from cervical cancer. This does not allow for the possibility that relative survival does not correspond to cause-specific survival nor for women who die from cervical cancer >5 years after diagnosis.

It has been suggested that healthy women are more likely to attend cervical screening (Dugue *et al*, 2014). Self-selection bias would result in an overestimation of the reduction in the risk of death following screening. We previously estimated that the impact of unrecorded confounders in the audit would be unlikely to change the results (for incidence) by more than ∼18% (RR=0.85; Castanon *et al*, 2014).

The estimates of the number of deaths from cervical cancer that could be prevented by regular screening or that are prevented by current screening rely on the assumption that the RRs calculated based on 5-year excess mortality approximate the RRs for cause-specific mortality. In this study, we group deaths by the age of cancer diagnosis not the age at death. Hence, screening (starting at age 25) will (most likely) increase the number of fatal cancers diagnosed at 25 years, even if it reduces (cumulative) cervical cancer mortality. Under the current screening programme, as the population age a large number of deaths that are not preventable by screening will occur after age 84 years, decreasing the relative benefit of screening overall.

Our model implicitly assumes that stage-specific survival is not affected by route to diagnosis (screen detected *vs* non-screen detected). If anything, it is likely that survival will be better in screen-detected women; if this corresponds to improved cure rates, it would cause our estimates to be conservative. Andrae *et al* (2012) found that even allowing for lead-time, screen-detected cancer was more likely to be cured than a symptomatic cancer diagnosed with the same stage. Similarly, Zucchetto *et al* (2013) found that stage-specific survival from cervical cancer among non-screen-detected women was significantly lower than among those with screen-detected cancer. These differences could simply be due to lead-time bias (stage 1A and 1B) or failure to differentiate stage II from stage III+ cervical cancer, in which case our approach would not be conservative.

The most direct method of evaluating the effect of screening on mortality requires individual-level data on survival following cervical cancer diagnoses, as well as the screening history for those individuals. We know of three such studies. Vicus *et al* (2014) analysed 1052 deaths from cervical cancer in Canada, and found that attending screening in the 3–36 months before cervical cancer diagnosis reduced mortality by 40–72%, depending on age, but that screening 37–60 or 61–120 months before diagnosis was not associated with any reduction in risk. Lonnberg *et al* (2013) analysed data on 506 women who had died from cervical cancer in 2000–2009. Overall, they reported a 66% reduction in mortality associated with attending a single programme screen. Both these studies found little effect of screening on mortality for young women (under age 40). Rustagi *et al* (2014) studied 39 deaths from cervical cancer in women aged 55–79 years, and found that screening in the 7 years before diagnosis was associated with a 74% reduction in mortality. Compared with the studies mentioned above, our much larger study found a greater impact on mortality. This may be a consequence of the high quality of cervical cytology in England (Cuzick *et al*, 2006).

In the absence of individual-level mortality and screening data, it is possible to use trend data to get a very rough estimate of the impact of screening on mortality by looking at the mortality rate before the introduction of cervical screening, and the current mortality rate, incorporating the average screening coverage over this time period. However, this would not allow for differences in treatment efficacy over this time, the differential impact for women who are regularly screened compared with women who are screened infrequently, nor for changes in the underlying rates of cervical cancer in the absence of screening. In addition, large improvements in quality assurance of the programme over the past 20 years have ensured equal access to expert care that has improved survival for all women with cervical cancer (Kitchener, 2008).

To conclude, we have shown that screening has an even larger impact on cervical cancer mortality than it has on incidence, and that if everyone attended screening regularly, 83% of cervical cancer deaths could be prevented, compared with 70% with current screening. These results are encouraging and should be used to promote, among women, regular attendance to screening and, among policy makers, the implementation of organised screening programmes in areas not yet covered.

## Figures and Tables

**Table 1:** Definition of regularly, irregularly, and not screened depending on age at case diagnosis

**Age at diagnosis (years)**	**25–34**	**35–64**	**65+**
Screening window under consideration	Age 25.5–28.5 years: screening 22.5–25.5 years Age 28.5–31.5 years: screening 22.5–28.5 years Age 31.5–35 years: screening 22.5–31.5 years	Screening 15 years before diagnosis	Screening at ages 50–64 years
Regularly screened	Age 25.5–28.5 years: screened 22.5–25.5 years Age 28.5–31.5 years: screened 22.5–25.5 and 25.5–28.5 years Age 31.4–35: screened 22.5–25.5, 25.5–28.5, and 28.5–31.5 years	Age 35–49 years: maximum interval between tests is ⩽3.5 years Age 50–64 years: maximum interval between tests is ⩽5.5 years	Maximum interval between tests is ⩽5.5 years
Irregularly screened	Age 28.5–31.5 years: screened 22.5–28.5 years, but not regularly Age 31.4–35 years: screened 22.5–31.5 years, but not regularly	Age 35–49 years: maximum interval is 3.5–7.5 years Age 50–64 years: maximum interval is 5.5–7.5 years	Maximum interval is 5.5–7.5 years
Very irregularly screened	NA	Maximum interval is 7.5–13 years	Maximum interval is 7.5–13 years
Not (or nearly never) screened	Age 25.5–28.5 years: not screened 22.5–25.5 years Age 28.5–31.5 years: not screened 22.5–28.5 years Age 31.5–35 years: not screened 22.5–31.5 years	No screen in window or maximum interval is >13 years	No screen in window or maximum interval is >13 years

**Table 2:** Odds ratios (OR) of a cervical cancer diagnosis by age, stage, and maximum interval between cervical screens

	**FIGO stage at diagnosis**											
		**Stage 1A**			**Stage 1B**			**Stage 2**			**Stage 3 or worse**	
**Screening history**	**% Cases**	**OR**	**95% CI**	**% Cases**	**OR**	**95% CI**	**% Cases**	**OR**	**95% CI**	**% Cases**	**OR**	**95% CI**
**Aged 25.5–34 years**		***n*=1780**			***n*=984**			***n*=214**			***n*=87**	
Not screened since age 22.5 years	31.5	1		40.2	1		54.7	1		56.3	1	
Regularly screened	35.7	0.76	0.65–0.88	31.7	0.59	0.48–0.71	28.5	0.28	0.17–0.43	32.2	0.16	0.06–0.37
Irregularly screened	32.8	0.95	0.80–1.12	28.0	0.72	0.58–0.88	16.8	0.39	0.25–0.60	41.4	0.27	0.13–0.56
OR linear trend		0.87	0.81–0.93		0.77	0.70–0.85		0.51	0.40–0.64		0.37	0.24–0.58
**Aged 35–64 years**		***n*=1921**			***n*=2231**			***n*=929**			***n*=748**	
Not screened or > 13 yearly	19.3	1		23.6	1		43.9	1		57.6	1	
<5.5 yearly	38.6	0.33	0.27–0.38	39.7	0.25	0.21–0.28	23.3	0.10	0.07–0.12	15.4	0.05	0.03–0.06
5.5–7.5 yearly	17.0	0.53	0.43–0.64	15.4	0.38	0.31–0.46	12.1	0.17	0.12–0.22	9.9	0.10	0.06–0.14
7.5–13 yearly	25.2	0.87	0.72–1.06	21.2	0.62	0.51–0.74	20.8	0.37	0.28–0.48	17.1	0.20	0.14–0.27
OR linear trend		0.66	0.63–0.70		0.63	0.60–0.67		0.46	0.42–0.50		0.37	0.33–0.42
**Aged 65–79 years**		***n*=66**			***n*=306**			***n*=356**			***n*=418**	
Not screened or > 13 yearly	36.4	1		35.3	1		39.3	1		47.6	1	
<5.5 yearly	47.0	0.31	0.14–0.67	44.4	0.39	0.28–0.54	40.4	0.32	0.24–0.44	31.8	0.19	0.14–0.26
5.5–7.5 yearly	6.1	0.30	0.08–1.04	8.5	0.53	0.31–0.91	7.9	0.29	0.18–0.48	7.4	0.23	0.14–0.37
7.5–13 yearly	10.6	0.29	0.10–0.84	11.8	0.59	0.37–0.96	12.4	0.62	0.39–1.01	13.2	0.61	0.40–0.94
OR linear trend		0.71	0.56–0.91		0.74	0.66–0.82		0.68	0.62–0.76		0.57	0.52–0.63

**Table 3:** Estimated case-fatality^a^ **rates in England by age and stage**

**Age group (years)**	**Stage 1A (%)**	**Stage 1B (%)**	**Stage 2 (%)**	**Stage 3+ (%)**
25.5–34	1.4	8.8	55.1	80.0
35–49	1.4	8.6	54.2	79.2
50–64	2.5	10.9	51.2	86.0
65–69	2.1	9.1	44.9	80.5
70–79	1.5	14.8	68.8	95.1

a Case-fatality is expressed as ‘1−S' where S is the 5-year relative survival.

**Table 4:** Estimated relative 5-year cervical cancer mortality under an absence of screening and regular screening compared with current screening

	**Relative 5-year mortality compared with current screening**					
**Age group at diagnosis (years)**	**In the absence of screening**	**For regular screening**	**Age group at death (years)**	**Observed deaths (average 2011–2014)**^a^	**Estimated deaths in the absence of screening**	**Estimated deaths with regular screening**
25–34^b^	1.96 (1.66–2.31)	0.68 (0.61–0.76)	25–39	103	202	70
35–49	4.13 (3.59–4.75)	0.42 (0.38–0.47)	40–54	175	721	73
50–64	5.30 (4.36–6.44)	0.35 (0.33–0.37)	55–69	199	1054	70
65–79	2.51 (2.18–2.90)	0.61 (0.58–0.65)	70–84	216	542	132
25–79	*3.64 (3.29–4.03)*^c^	*0.50 (0.48–0.52)*^c^	25–84	692	2519	345
All ages	*3.30 (2.92–3.72)*^c^	*0.56 (0.55–0.58)*^c^	All ages	796	2623	449

a Reported by ONS (Office for National Statistics, 2015).

b Note that we have included women aged 25–25.5 years so as to estimate the effect of the screening programme as a whole. OR for women aged 25.5–34 and 24.5–34 years are presented in the Supplementary Table.

c Estimated as the ratio of the estimated *vs* observed deaths. 95% CI calculated were obtained from the combined variance of the individual age groups.

**Table 5:** Association between screening and cervical cancer incidence and mortality

**Age group (years)**	**Screening history**	**Direct OR for incidence**^a^	**Indirect RR for incidence**^b^	**Indirect RR for mortality**
25.5–34	Regularly screened	0.63 (0.56–0.70)	0.61 (0.54–0.68)	0.27 (0.21–0.34)
	Irregularly screened	0.77 (0.68–0.87)	0.76 (0.67–0.86)	0.37 (0.29–0.46)
	Never screened	1.00	1.00	1.00
35–64	<5.5 yearly	0.18 (0.16–0.20)	0.18 (0.16–0.19)	0.08 (0.07–0.09)
	5.5–7.5 yearly	0.29 (0.26–0.33)	0.28 (0.25–0.32)	0.14 (0.12–0.17)
	7.5–13 yearly	0.51 (0.46–0.57)	0.49 (0.41–0.60)	0.28 (0.23–0.33)
	Not screened or >13 yearly	1.00	1.00	1.00
65–79	<5.5 yearly	0.28 (0.24–0.34)	0.26 (0.22–0.32)	0.24 (0.20–0.30)
	5.5–7.5 yearly	0.31 (0.24–0.42)	0.31 (0.23–0.42)	0.28 (0.19–0.39)
	7.5–13 yearly	0.57 (0.44–0.74)	0.57 (0.43–0.75)	0.60 (0.46–0.77)
	Not screened or >13 yearly	1.00	1.00	1.00

a Without reference to stage.

b Combining stage-specific estimates.
