# Supplementary material for: Impact of cervical screening on cervical cancer mortality: estimation using stage-specific results from a nested case–control study
Source: Br J Cancer. 2016 Sep 15;115(9):1140–6. doi: 10.1038/bjc.2016.290 (PMC5117785; doi:10.1038/bjc.2016.290)
Supplement: Supplementary Table [file bjc2016290x1.doc]

| Supplementary Table. Sensitivity analysis varying the main assumptions in the analysis. | | | | | | | | | |  | |  |  | | |
| --- | --- | --- | --- | --- | --- | --- | --- | --- | --- | --- | --- | --- | --- | --- | --- |
|  | **25.5 – 34** | | **25.5 – 34** | | **35 – 49** | | **35 – 49** | | **50-64** | | **50-64** | | | **65-79** | **65-79** |
|  | **RR incidence** | | **RR mortality** | | **RR incidence** | | **RR mortality** | | **RR incidence** | | **RR mortality** | | | **RR incidence** | **RR mortality** |
| **Using the assumptions made in the manuscript** | | | | | | |  | |  | |  | | |  |  |
| 15-year window. Stage 1A has mortality of 1B | 1.29 | | 2.20 | | 2.56 | | 4.13 | | 4.15 | | 5.30 | | | 2.42 | 2.51 |
|  | (1.20 to 1.39) | | (1.77 to 2.75) | | (2.37 to 2.77) | | (3.59 to 4.75) | | (3.63 to 4.74) | | (4.36 to 6.44) | | | (2.17 to 2.71) | (2.18 to 2.90) |
| **Screening window changes** | | | | |  | |  | |  | |  | | |  |  |
| 8-year window |  | |  | | 2.79 | | 3.86 | | 3.01 | | 3.49 | | | 2.14 | 2.29 |
|  |  |  |  |  | (2.60 to 2.99) | | (3.42 to 4.36) | | (2.61 to 3.47) | | (2.93 to 4.16) | | | (1.83 to 2.50) | (1.92 to 2.73) |
| 12-year window |  | |  | | 2.87 | | 4.14 | | 3.96 | | 4.75 | | | 2.36 | 2.47 |
|  |  |  |  |  | (2.67 to 3.07) | | (3.66 to 4.69) | | (3.49 to 4.48) | | (3.99 to 5.67) | | | (2.10 to 2.65) | (2.14 to 2.86) |
| Screened in the last two intervals (none ,1 or 2) |  | |  | | 2.76 | | 3.78 | | 3.95 | | 4.82 | | | 2.43 | 2.55 |
|  |  |  |  |  | (2.50 to 3.05) | | (3.14 to 4.55) | | (3.55 to 4.40) | | (4.13 to 5.62) | | | (2.18 to 2.71) | (2.28 to 2.87) |
| Screened in the last two intervals (None, second, first or both intervals) |  | |  | | 2.76 | | 3.79 | | 4.00 | | 4.92 | | | 2.46 | 2.57 |
|  |  |  |  |  | (2.57 to 2.97) | | (3.35 to 4.28) | | (3.50 to 4.59) | | (4.04 to 6.00) | | | (2.18 to 2.78) | (2.19 to 3.00) |
| **Survival of Stage 1A(unscreened)** | | | | | | | | | | | | | | | |
| Same as 1A |  | | 1.80 | |  | | 3.90 | |  | | 5.19 | | |  | 2.49 |
|  |  |  | (1.38 to 2.36) | |  |  | (3.37 to 4.52) | |  |  | (4.26 to 6.33) | | |  | (2.16 to 2.88) |
| **Allocation of women with missing FIGO stage** | | | | | |  | |  | |  | |  |  | | |
| No reallocation | 1.27 | | 2.28 | | 2.50 | | 4.17 | | 4.10 | | 5.32 | | | 2.40 | 2.50 |
|  | (1.19 to 1.37) | | (1.85 to 2.81) | | (2.31 to 2.69) | | (3.64 to 4.78) | | (3.60 to 4.68) | | (4.39 to 6.45) | | | (2.14 to 2.69) | (2.16 to 2.88) |
| All 2 | 1.33 | | 2.13 | | 2.67 | | 4.1 | | 4.13 | | 5.06 | | | 2.39 | 2.42 |
|  | (1.22 to 1.44) | | (1.76 to 2.59) | | (2.45 to 2.92) | | (3.57 to 4.71) | | (3.62 to 4.71) | | (4.25 to 6.03) | | | (2.13 to 2.68) | (2.12 to 2.78) |
| All 3+ | 1.38 | | 2.37 | | 2.79 | | 4.44 | | 4.48 | | 5.62 | | | 2.53 | 2.62 |
|  | (1.17 to 1.63) | | (1.50 to 3.75) | | (2.52 to 3.08) | | (3.70 to 5.33) | | (3.82 to 5.26) | | (4.48 to 7.04) | | | (2.24 to 2.86) | (2.24 to 3.06) |
| Proportionally 1A, 1B, 2 & 3+ | 1.27 | | 2.28 | | 2.50 | | 4.17 | | 4.10 | | 5.32 | | | 2.42 | 2.52 |
|  | (1.19 to 1.37) | | (1.85 to 2.81) | | (2.31 to 2.69) | | (3.64 to 4.78) | | (3.60 to 4.68) | | (4.39 to 6.45) | | | (2.16 to 2.71) | (2.18 to 2.90) |
| **Young women** |  |  | |  | |  | |  | |  | |  |  | | |
| Including 24.5 to 34 | 1.02 | | 1.84 | |  | |  | |  | |  | | |  |  |
|  | (0.96 to 1.08) | | (1.60 to 2.13) | |  |  |  |  |  |  |  |  |  |  |  |
